# Parkinson's in the bone

**DOI:** 10.1186/s13578-021-00702-5

**Published:** 2021-11-05

**Authors:** Lei Xiong, Jin-Xiu Pan, Hao-han Guo, Lin Mei, Wen-Cheng Xiong

**Affiliations:** 1 Department of Neurosciences, School of Medicine, Case Western Reserve University, Cleveland, OH 44106 USA; 2 Louis Stoke VA Medical Center, Cleveland, OH 44106 USA

**Keywords:** Parkinson's disease, Bone homeostasis, Osteoporosis

## Abstract

Patients with Parkinson’s disease (PD) exhibit systemic deficits, including arthritis and osteoporosis-like symptoms. However, the questions, how the deficits in periphery organs or tissues occur in PD patients, and what are the relationship (s) of the periphery tissue deficits with the brain pathology (e.g., dopamine neuron loss), are at the beginning stage to be investigated. Notice that both PD and osteoporosis are the products of a complex interaction of genetic and environmental risk factors. Genetic mutations in numerous genes have been identified in patients either with recessive or autosomal dominant PD. Most of these PD risk genes are ubiquitously expressed; and many of them are involved in regulation of bone metabolism. Here, we review the functions of the PD risk genes in regulating bone remodeling and homeostasis. The knowledge gaps in our understanding of the bone-to-brain axis in PD development are also outlined.

## Introduction

Parkinson's disease (PD) is the 2nd most common neurodegenerative disease, clinically characterized as a movement disorder. Since Dr. James Parkinson initially describes the symptoms of “Shaking Palsy”, and Dr. Charcot re-named it as “Parkinson’s Disease” (PD) [1–3], nearly two centuries have passed. Until now, PD remains to be a terminal degenerative disease without effective cure therapy. Interestingly, PD was considered as a non-genetic disease long time ago, because epidemiological studies showed evidence to link PD with the environmental factors such as neurotoxins or viral infection [4, 5]. For examples, drug users exposed to 1-methyl-4-phenyl-1,2,5,6-tetrahydropyridine (MPTP) show parkinsonian-like features, and a pandemic influenza virus is found to be strongly associated with post-encephalitic parkinsonism [6]. In 1996, it marked the starting point for PD genetic studies, which mapped and subsequently identified mutations in *SNCA* (α-synuclein) or *PARK1* gene to be responsible for PD [7, 8]. In the following years, additional mutations related to PD, such as *PINK1*, *PRKN*, *LRRK2*, and *VPS35*, have been identified [9, 10]. To date, more than 20 autosomal dominant and recessive genetic mutations have been found to be involved in the pathogenesis of PD [11, 12].

It is also of interest to note that PD was considered as a disease with systemic deficits, including arthritic joint pain, constipation, muscle weakness and rigidity, and increased incidence of osteoporosis and bone fractures, according to Drs. Parkinson and Charcot' initial descriptions [2, 3]. However, since the discovery of the dopamine neuron loss in PD patients' brain in 1960s [13], the central dopamine hypothesis has been dominant in the field. Recent molecular, biological, and genetic studies suggest that the PD risk genes are expressed not only in the brain, but also in periphery organs/tissues, including bone cells; and mouse models expressing or suppressing the PD risk gene could exhibit phenotypes not only in the brain, but also in periphery organs/tissues [14, 15]. These observations have thus led scientists to re-think the view that PD is a systemic disorder.

Bone remodeling is a dynamic process essential for maintenance of skeletal integrity and bone homeostasis, in which old bone is destructed by bone-resorbing osteoclasts (OCs) and subsequently replaced with new bone by bone-forming osteoblasts (OBs) [16]. The imbalance of bone formation and resorption could result in metabolic bone diseases such as osteoporosis. PD and osteoporosis are two conditions affecting a substantial portion of the elderly population [17]. PD patients usually have bone and joint problems, and have a high risk for fractures, which are believed to be induced by falls due to decreased mobility, postural instability, neurological impairment and reduced bone mass [18]. Both PD and osteoporosis are the product of a complex interaction of genetic and environmental factors. Many genetic mutations identified in PD patients have been found to regulate not only PD's brain pathology, but also bone metabolism (Table 1). Below, we summarize the functions of these PD risk genes in regulating bone remodeling and bone homeostasis.

**Table 1:** PD risk genes that affect bone metabolism

Gene (symbol)	Chromosomal location	Inheritance	Biological function	Phenotype
*FBXO7* *(PARK15)*	4q22.1	AD	Regulate proteasome-independent ubiquitination of NRAGE	Increase BMP4-mediated signaling in HEK293 cells [40]
*HTRA2* *(PARK13)*	2p13.1	AD	Enhanced the stability of TNF receptor-associated factor 2;	Activation of the inflammatory response in mouse arthritis model [79]
*LRRK2* *(PARK8)*	12q12	AD	Inhibit canonical Wnt signaling, decrease the levels of transcriptionally active β-catenin	Lrrk2-KO mice shown increased tibial cortical bone strength [47]
*NR4A2* *(NURR1)*	2q22-q23	Risk factor	NR4A2 expression was induced by PTH-cAMP/PKA pathway; Enhanced the transcription of FGF23; Activate the OPN promoter	Inhibit bone resorption in mouse model [71–73]
*DJ-1* *(PARK7)*	1p36.23	AR	Decrease intracellular ROS concentration and increase the activity of SHP-1 during osteoclastogenesis; Activate FGF receptor-1 signaling	Increase bone mass via negatively regulation of osteoclastogenesis and promotes osteoblasts differentiation [31, 32]
*PINK1* *(PARK6)*	1q36.12	AR	Regulate mitophagy	Play a protective role in bone impairment [24]
*PRKN* *(PARK2)*	6q26	AR	Regulate mitophagy; promoteβ-catenin expression and autophagy	Play a protective role in bone impairment; Promote osteoblastic differentiation and accelerate bone healing in mouse model [24–26]
*SNCA* *(PARK1; PARK4)*	4q22.1	AD	a key mediator of the expression of specific network modules and the skeletal response to estrogen deficiency	Required for OVX-induced bone loss in mice [76, 77]
*UCHL1* *(PARK5)*	4p13	AD	A component of the ubiquitin proteasome system	Regulates bone mineralization during osteogenesis in mouse model [37]
*VPS35* *(PARK17)*	16q11.2	AD	Terminate PTH signaling in OBs; Inhibit RANKL signaling in OCs	Promote bone formation and inhibit bone resorption in mouse model [57, 58]

*AD* autosomal dominant, *AR* autosomal recessive

### Autosomal recessive PD risk genes in autophage, mitochondrial function, and bone homeostasis

#### PINK1 and PRKN

PINK1, stands for PTEN induced putative kinase 1 (PINK1), is also called PARK6 [19]. PRKN, also named as Parkin or PARK2, is an E3 ubiquitin ligase [20]. Genetic studies have identified mutations in both PINK1 and PRKN genes in patients with recessive PD [21, 22]. Cell biological studies have suggested that both proteins form a complex, and PRKN is one of substrates of PINK1 [20]; defects in PINK1 and Parkin cause an impairment in mitophage mediated clearance of damaged mitochondria, and thus an accumulation of dysfunctional mitochondria, leading to the loss of DA neurons with age [23].

Interestingly, PINK1 and Parkin-mediated mitophagy is reported to play a protective role in bone impairment induced by aluminum exposure [24]. Parkin-deficient mice exacerbated bone impairment, mitochondrial damage, and oxidative stress under aluminum exposure [24]. Zhang et al. have found that parkin could meditate osteoblastic differentiation of BMSCs via β-catenin and autophagy signaling [25]. Upregulation of parkin could promote β-catenin expression and autophagy, enhancing expression of osteo-specific markers [25]. Moreover, parkin-overexpression accelerates bone healing in a tibial fracture model [25]. Furthermore, it is reported that p53 and parkin co-regulate mitophagy in bone marrow mesenchymal stem cells to promote the repair of early steroid-induced osteonecrosis of the femoral head (ONFH) [26].

#### PARK7 (DJ-1)

PARK7 also known as DJ-1, is a 189 amino acid protein that is widely expressed in different organs/tissues [27]. PARK7 is linked to an early onset of autosomal recessive PD via homozygous deletion and loss of function mutation of *PARK7* gene [28]. Cell biological studies have suggested PARK7's function in regulating mitochondrial function and preventing ROS (free oxygen species) production [29, 30].

Notably, a recent study, by Kim et al., has uncovered a pivotal role of PARK7 in regulating bone homeostasis [31]. PARK7 (DJ-1) appears to play a critical role in negative regulation of osteoclastogenesis, as well as bone-associated pathology [31]. PARK7 deficient mice exhibit a decrease in bone volume, but an increase in OC numbers, which are likely due to the increased intracellular ROS concentration, and RANKL-induced signaling [31]. In addition to its effects on osteoclastogenesis, PARK7 has been found to stimulate the differentiation of human mesenchymal stem cells to osteoblasts; and to induce angiogenesis in endothelial cells through activation of fibroblast growth factor receptor-1 signalling [32]. Extracellular application of PARK7 could enhance bone regeneration by stimulating the formation of blood vessels and new bones in the rodent model of bone fracture repair [32].

### Autosomal recessive PD risk genes in protein ubiquitination, degradation, and bone homeostasis

#### UCHL1 (PARK5)

Ubiquitin C-terminal hydrolase L1 (UCHL1) is a component of the ubiquitin proteasome system. Despite the controversy, UCHL1 S18Y variant is reported to be a risk factor for PD [33–36]. Shim et al. found that UCHL1 is expressed in osteoblasts, osteoclasts, and hematopoietic precursor cells of bone marrow in the metaphysis and diaphysis of the femora. In addition, gracile axonal dystrophy (gad) mice, which lack UCHL1 expression, shown reduced bone mineral density (BMD) rate in the metaphysis and diaphysis of the femora, suggest a critical role of UCHL1 in regulating bone mineralization during osteogenesis [37].

#### FBXO7

As a member of the F-box protein (FBP) family, FBXO7 (F-box only protein 7) is a substrate-recognition component of Skp1-cullin-F-box protein ubiquitin E3 ligase, which function in phosphorylation-dependent ubiquitination [38]. Mutations in FBXO7 gene are identified in patients closely associated with progression of the autosomal recessive form of familial PD [39].

FBXO7 is reported to affect bone morphoenetic protein 4 (BMP4)-mediated signaling through proteasome-independent ubiquitination of NRAGE and augments formation of downstream signaling components [40]. However, this study is mainly carried out in vitro, and it remains to be investigated whether it affects bone remodeling and homeostasis in vivo.

### Autosomal dominant PD risk genes in Wnt/β-catenin signaling and bone homeostasis

#### Wnt/β-catenin signaling

**Fig. 1:**
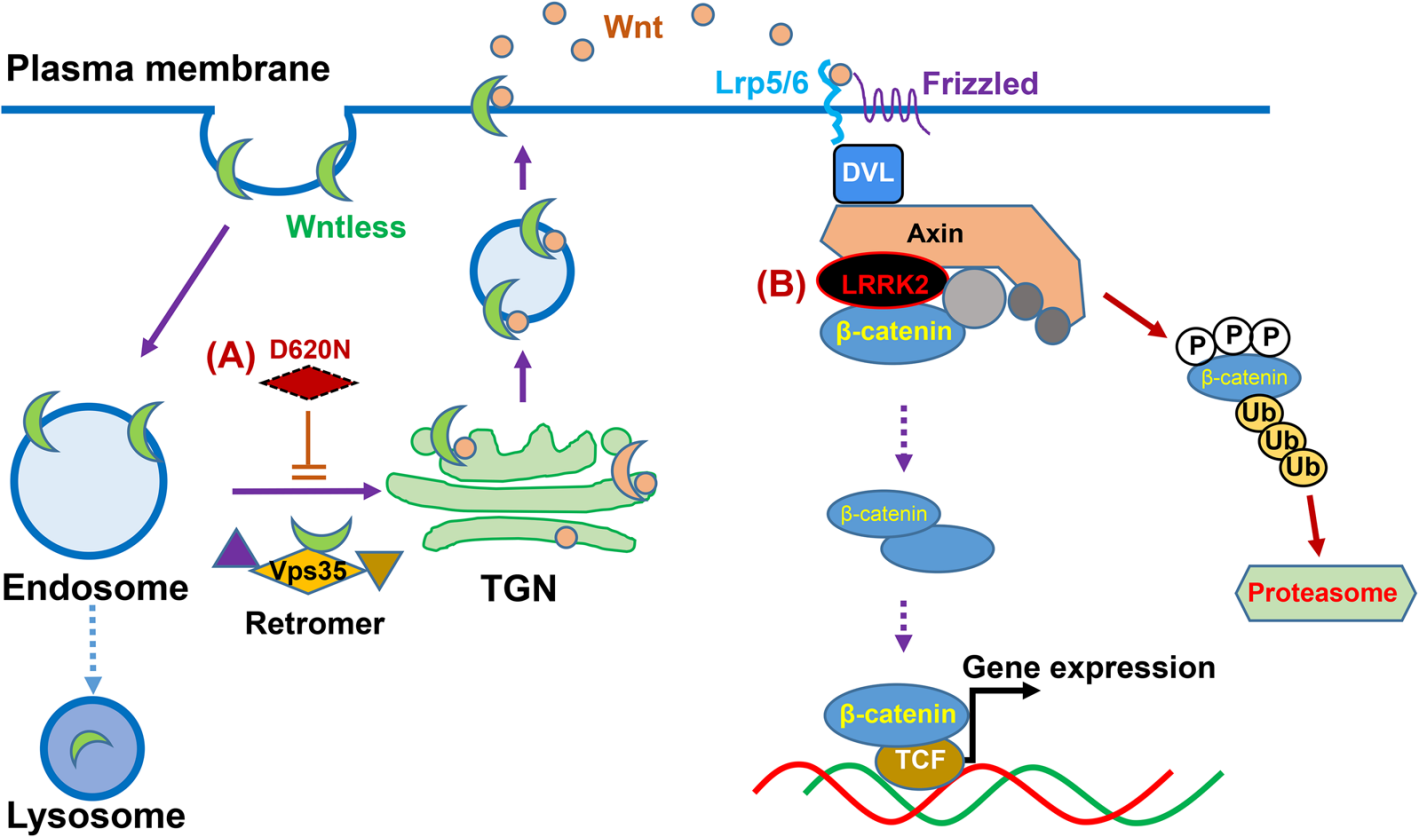
PD risk genes that regulate Wnt/β-catenin signaling and bone homeostasis. **A** Retromer complex (including Vps35) promotes Wnt secretion by recycling Wntless from endosomes to the trans-Golgi network (TGN). VPS35-D620N, a mutant in PD patients, inhibits this event, reducing Wnt secretion. **B** LRRK2 binds to the β-catenin destruction complex. Gain of LRRK2 function/PD pathogenic LRRK2 mutants repress β-catenin signaling; and loss of LRRK2 increases canonical Wnt/b-catenin signaling

The canonical Wnt/β-catenin signaling is essential for regulating bone-mass homeostasis [16, 41]. The secretion of Wnt depends on the wntless receptor [42–44] (Fig. 1). The binding of Wnt ligands to a dual-receptor complex of frizzled and Lrp5/6 results in the accumulation of cytoplasmic β-catenin and translocation of β-catenin into the nucleus to regulate gene expression (Fig. 1). This pathway is necessary for commitment of mesenchymal stem cells to the OB lineage, OB precursor cell proliferation and differentiation, and OC genesis and activation [16, 41, 45].

#### LRRK2 (PARK8)

Leucine rich repeat kinase 2 (LRRK2) mutations account for up to 40% of PD cases in some populations, and elicit symptoms and brain pathologies resembling idiopathic PD [46]. By using Lrrk2 knockout mice, Berwick et al. investigated the effect of loss of Lrrk2 on canonical Wnt signaling in vitro and in vivo. They found that loss of Lrrk2 causes a dose-dependent increase in the levels of transcriptionally active β-catenin in the brain, while over-expressed LRRK2 binds and represses β-catenin, suggesting Lrrk2 may act as part of the β-catenin destruction complex. Lrrk2-KO could also increase tibial cortical bone strength so that alters tibial bone architecture, decreasing the predicted risk of fracture [47]. Interestingly, many Lrrk2 mutants identified in PD patients appear to gain of LRRK2's functions [48, 49], and thus suppressing wnt/β-catenin signaling (Fig. 1B).

#### VPS35 (PARK17)

The vacuolar protein sorting ortholog 35 (VPS35) is a key component of the retromer, which is responsible for selective retrieval of transmembrane cargo proteins from endosome to trans-Golgi apparatus (Fig. 1A). VPS35 is also called PARK 17, because mutations in Vps35/Park 17 locus, such as D620N and R524W, have been unambiguously identified to cause PD in multiple individuals and families worldwide [50–52]. Thus, dysfunctional VPS35/retromer has been recently emerged as a new risk factor for late-onset, autosomal dominant familial PD.

Notice that VPS35/retromer is widely expressed in many tissues and cell types, not only in dopamine neurons [53–56], but also in bone cells, such as osteoblasts and osteoclasts [57, 58]. Vps35 hemizygous deficient mice exhibit osteoporotic deficits with increased bone resorption and decreased bone formation [57, 58]. Interestingly, Xia et al. have found that RANK (receptor activator of NF-κB) in macrophages or osteoclast-lineage cells is a cargo protein of VPS35/retromer [57]; and loss of VPS35's function in macrophages or osteoclast-lineage cells results in increased RANK surface distribution, enhanced RANKL sensitivity, sustained RANKL-RANK signaling, and thus increasing hyper-resorptive osteoclast formation [57].

In addition to the increased osteoclast mediated bone resorption, mice with Vps35 hemizygous deficiency or the osteoblast selective VPS35 conditionally knocking out display decreased osteoblast mediated bone formation [57, 58], another key cellular process underlying the osteoporotic bone loss. In light of numerous reports that demonstrate that Wntless, a sorting receptor, work together with the retromer complex to promote the secretion of Wnt family proteins [59–63] (Fig. 1); and the loss of Wntless impairs Wnt secretion, reducing Wnt/b-catenin signaling and bone formation [64, 65], it is likely that VPS35 regulating Wntless trafficking and function in osteoblast-lineage cells may account for their function in promoting osteoblast mediated bone formation. This view is in line with a report by Chiu et al., who have found that the VPS35-D620N mutation causes the malfunction of Vps35 and impairs activity of Wnt/β-catenin pathway in substantia nigra pars compacta dopaminergic neurons [66] (Fig. 1A). However, this view requires further investigation in osteoblast lineage cells.

### PD risk genes in PTH signaling and bone homeostasis

#### PTH signaling

**Fig. 2:**
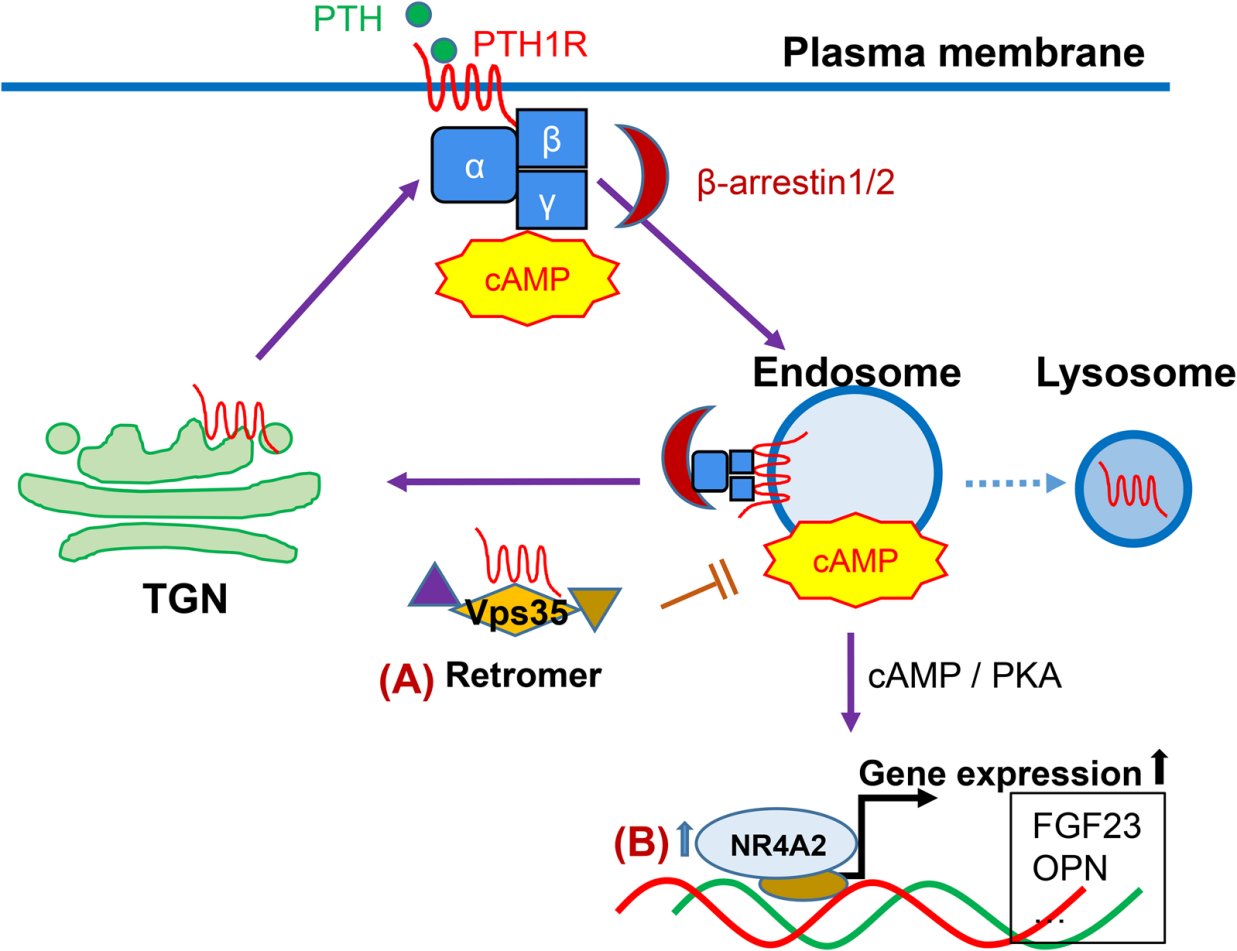
PD risk genes that regulate PTH1R signaling and bone homeostasis. **A** The PD risk gene, VPS35 (a key component of Retromer) plays an important role in regulating PTH1R trafficking, turning off PTH signaling, and promoting its catabolic function. **B** The PD risk gene, NR4A2, is up-regulated by PTH-PTH1R-cAMP signaling, which then acts as a transcriptional activator to induce gene expression such as fibroblast growth factor 23 (FGF23) and osteopontin (OPN)

The recombinant parathyroid hormone (PTH) is the only therapy for postmenopausal osteoporosis that increase bone mass. Intermittent treatment with PTH[1–34] promotes recruitments of both osteoblasts and osteoclasts, resulting in a net bone-gain, but its continued treatment leads to more osteoclast activation with a net bone-loss [67]. Binding of PTH and PTH receptor (PTH1R) initiates intracellular cyclic adenosine monophosphate (cAMP) signaling that is dynamically regulated [68] (Fig. 2). Below, we highlight the PD risk genes' function in regulating PTH signaling and bone homeostasis.

#### VPS35

Notice that in addition to Wntless and RANK, PTH1R is also identified as a cargo of VPS35/retromer, and the PTH1R signaling is dynamically regulated by the retromer complexes [58] (Fig. 2A). Vps35 knockdown in kidney cells [68] and osteoblasts [58] resulted in a slight elevation and extended cAMP response to PTH. Xiong et al. further investigate VPS35's function in regulating PTH signaling in vivo. Specific Vps35 knockdown in the osteoblast lineage cells, using osteocalcin-targeted-Cre, results in mildly lowered bone mass in the primary spongiosa, but show a greater increase in bone mass in the primary spongiosa in response to PTH, as compared to controls [58]. They further provide evidence for Vps35 to regulate PTH1R trafficking, to turn off PTH signaling, and to promote its catabolic function in culture and in mice [58]. Additionally, Xiong et al. have identified phosphatase 1 regulatory subunit 14C (PPP1R14C), an inhibitory subunit of PP1 phosphatase, as a VPS35 binding partner, to be involved in VPS35/retromer termination of the endosomal PTH1R signaling [58]. However, whether and how VPS35 mutants (e.g., D620N) alter PTH signaling and bone homeostasis remain to be investigated.

#### NR4A2 (Nurr1)

Nuclear receptor subfamily 4 group A member 2 (NR4A2), also known as Nurr1, is a member of the nuclear receptor family of intracellular transcription factors [69]. It is mainly expressed in the central nervous system, but it is also detectable in peripheral tissues such as bone cells. NR4A2 is involved in the pathogenesis of diseases that need dopamine transmission such as PD [70]. Tetradis et al. found that PTH could induce the expression of NR4A2 in primary mouse osteoblasts, which is mediated primarily through the cAMP/PKA pathway [71] (Fig. 2B). The increased NR4A2 then enhances the PTH-induced transcription of FGF23 (fibroblast growth factor 23), a critical growth factor for skeleton development [72]. NR4A2 is also found to mediate PTH-induced expression of osteopontin (OPN) [73]. NR4A2 appears to be transcriptional activator for OPN expression, as it binds and activates the OPN promoter in osteoblastic cells in a synergistic fashion (Fig. 2B), and thus regulate bone homeostasis [73].

### Other autosomal dominant PD risk genes in bone homeostasis

#### SNCA

Alpha-synuclein is a small 140 amino polypeptide encoded by *SNCA* gene that is a major component of Lewy body (LB) inclusions in PD patients [74, 75]. Alpha-synuclein is highly expressed in neural tissue, osteoblasts, erythroblasts, macrophages, and adipose tissue [76]. As an autosomal dominant gene, *SNCA* was found to regulate bone network homeostasis and ovariectomy-induced bone loss [76]. Calabrese et al. used a mouse model of postmenopausal bone loss (ovariectomy-induced bone loss) and variation in gene expression generated by the divergent genetic backgrounds of inbred mouse strains to construct a bone co-expression network in intact and ovariectomized mice, they identified a module of genes whose expression is associated with OVX-induced bone loss, and alpha-synuclein (*Snca*), is a key mediator of the expression of specific network modules and the skeletal response to estrogen deficiency. In addition, *Snca* deficiency protects mice from OVX-induced bone loss, the bone loss due to OVX was significantly less in Snca^−/−^ mice than littermate controls [76]. However, using the Prrx1Cre to conditionally delete *Snca* in osteoprogenitor cells, Figueroa et al. show that deletion of *Snca* in Prrx-Cre( +) cells causes partial loss of function in the central nervous system but does not affect OVX-induced bone loss [77], implicating that *Snca* may regulate bone remodeling in an indirect manner. Further analysis is necessary to address this issue.

#### HTRA2 (OMI; PARK13)

HtrA serine peptidase 2 (HTRA2), also known as OMI, is a mitochondrially-located serine protease which is involved in PD [78]. Rheumatoid arthritis (RA) is an autoimmune inflammatory disease characterized by the destruction of cartilage and bone. HtrA2 was reported to participated in the activation of the inflammatory response in a collagen-induced arthritis model. HtrA2 modulated inflammatory responses in bone marrow-derived macrophages (BMDMs) by controlling TNF receptor-associated factor 2 (TRAF2) stability in a collagen-induced arthritis mouse model [79].

## Conclusion and future questions

Osteoporosis in PD patients has not been extensively studied. On one hand, decreased mobility, abnormal posture, as well as falling increase the risk of fractures. On the other hand, many PD risk genes have been found to be directly involved in the regulation of bone remodeling. Further investigating this issue may reveal new insights into the strategy developments for the clinical diagnosis and treatment of PD. A clinical fracture risk evaluation and bone densitometry to detect osteoporosis in newly identified PD patients may be useful for our further understanding their association. The medication used for the long-term treatments of PD may need safety studies for bone preservation, as we do not know whether these medications have any effect on bone health. PD and osteoporosis risk genes may have similar mechanisms in pathogenicity. However, further investigating their functions in bone may be helpful in the design of the new targeted drug development that can simultaneously treat both PD and osteoporosis.

Notice that many PD risk genes (such as *ATP13A2, EIF4G1, GBA, VPS13C*, and so on)' functions in bone remodeling and homeostasis have not been investigated, although they are widely expressed in various tissues and have important regulatory effects on cell metabolism such as autophagy, immune response, mitochondrial biology, lysosomal dysfunction (e.g. *GBA*, *ATP13A2*) and endocytic pathway [80, 81]. For example, the *GBA* gene encodes a lysosomal enzyme β-glucocerebrosidase with an important role in glycolipid metabolism. Loss-of-function mutations in β-glucocerebrosidase cause an accumulation of glucocerebroside that results in a wide spectrum of symptoms involving the liver, blood, bone marrow, spleen, lungs, and the nervous system, known as Gaucher disease [82]. Gaucher disease can weaken bone, increasing the risk of painful fractures. It can also interfere with the blood supply to your bones, which can cause portions of the bone to die [83]. Thus, in addition to brain, it is of importance to investigate the functions of these genes in periphery tissues or organs, including bone.

## Data Availability

Not applicable.

## Abbreviations

BMD: Bone mineral density
BMDMs: Bone marrow-derived macrophages
BMP4: Bone morphoenetic protein 4
BMSCs: Bone marrow stem/stromal cells
cAMP: Cyclic adenosine monophosphate
FBXO7: F-box only protein 7
FGF23: Fibroblast growth factor 23
HTRA2: HtrA serine peptidase 2
LB: Lewy body
LRRK2: Leucine rich repeat kinase 2
MPTP: 1-Methyl-4-phenyl-1,2,5,6-tetrahydropyridine
NR4A2: Nuclear receptor subfamily 4 group A member 2
OBs: Osteoblasts
OCs: Osteoclasts
ONFH: Osteonecrosis of the femoral head
PD: Parkinson’s disease
PINK1: PTEN induced putative kinase 1
PPP1R14C: Phosphatase 1 regulatory subunit 14C
PTH: Parathyroid hormone
RA: Rheumatoid arthritis
RANK: Receptor activator of NF-κB
ROS: Free oxygen species
TRAF2: TNF receptor-associated factor 2
UCHL1: Ubiquitin C-terminal hydrolase L1
VPS35: Vacuolar protein sorting ortholog 35
